# Visual analytics in healthcare education: exploring novel ways to analyze and represent big data in undergraduate medical education

**DOI:** 10.7717/peerj.683

**Published:** 2014-11-25

**Authors:** Christos Vaitsis, Gunnar Nilsson, Nabil Zary

**Affiliations:** 1 Department of Learning, Informatics, Management and Ethics, Karolinska Institutet, Stockholm, Sweden; 2 Department of Neurobiology, Care Sciences and Society, Karolinska Institutet, Stockholm, Sweden

**Keywords:** Visual analytics, Big data, Medical education, Data analysis, Curriculum mapping, Information visualization, Medical informatics

## Abstract

**Introduction.** The big data present in the medical curriculum that informs undergraduate medical education is beyond human abilities to perceive and analyze. The medical curriculum is the main tool used by teachers and directors to plan, design, and deliver teaching and assessment activities and student evaluations in medical education in a continuous effort to improve it. Big data remains largely unexploited for medical education improvement purposes. The emerging research field of visual analytics has the advantage of combining data analysis and manipulation techniques, information and knowledge representation, and human cognitive strength to perceive and recognize visual patterns. Nevertheless, there is a lack of research on the use and benefits of visual analytics in medical education.

**Methods.** The present study is based on analyzing the data in the medical curriculum of an undergraduate medical program as it concerns teaching activities, assessment methods and learning outcomes in order to explore visual analytics as a tool for finding ways of representing big data from undergraduate medical education for improvement purposes. Cytoscape software was employed to build networks of the identified aspects and visualize them.

**Results.** After the analysis of the curriculum data, eleven aspects were identified. Further analysis and visualization of the identified aspects with Cytoscape resulted in building an abstract model of the examined data that presented three different approaches; (i) learning outcomes and teaching methods, (ii) examination and learning outcomes, and (iii) teaching methods, learning outcomes, examination results, and gap analysis.

**Discussion.** This study identified aspects of medical curriculum that play an important role in how medical education is conducted. The implementation of visual analytics revealed three novel ways of representing big data in the undergraduate medical education context. It appears to be a useful tool to explore such data with possible future implications on healthcare education. It also opens a new direction in medical education informatics research.

## Introduction

### Medical education

Continuous efforts to improve medical education today are driven by the need to create competent health professionals able to meet rising healthcare demands. One approach has been to react to observed deficiencies in healthcare that were linked to unsatisfactory required competencies (Frenk et al., 2011). Cho et al. (2011) highlighted the difficulties that physicians had in keeping pace with the growing medical literature and therefore proposed to include a journal club in undergraduate medical education to acquire and instill at an early stage the ability to review scientific literature critically, a skill vital for a future physician using evidence-based medicine. A review of the literature revealed few studies reporting on improving medical education based on educational data such as assessment results and evaluation data (Gathright et al., 2009). Corrin & Olmos (2010) analyzed data from medical education, and more particularly reflective records from clinical experiences, through an online clinical log system and showed that it could help medical faculties to enhance the alignment between medical students’ clinical experiences and the curriculum taught. In another study, previously unperceived discrepancies between taught and the assessed curriculum in medical program were revealed using a web-based learning objectives database (Hege et al., 2010).

### Big data

Big data is broadly defined as the existence or emergence of datasets of such magnitude that it is beyond commonly-used tools’ (mainly databases) abilities to store, manipulate, and analyze. The public, commercial, and social sectors receive and produce vast amounts of data from different sources and in different forms every day, hour, and minute. The size of the data involved runs to terabytes or even petabytes and exceeds both hardware and human limits of easy processing, so it is termed big data. However, this term is sometimes arbitrarily assigned to large-sized data even when it may not be applicable, and what big data actually is can and does vary from sector to sector and more specifically between services within a sector (Manyika et al., 2011).

The size of data is in fact only one characteristic that qualifies data as big data. Other characteristics that define big data beyond size or volume are variety and velocity. The former refers to the different types of data and different sources from which it is collected in structured and unstructured forms, while the latter refers not only to the speed at which data is produced but also to the time required to process it, whether in real time or occasionally (Eaton et al., 2012).

### Big data in higher education

Higher education is one of the domains where data frequently collected from students’ usage and interaction, course information, and other academic data like administration and curricula is of such size and type that special techniques must be applied to discover new knowledge (Romero & Ventura, 2007).

It has been reported that big data in higher education has the potential to enable the development of insights “regarding student performance and learning approaches” and affect positively key areas like student’s actual performance according to the taught curriculum (West, 2012). Additionally, big data and analytics in higher education have recently been seen as holding great potential to promote actions concerning “administrative decision-making and organizational resource allocation”, early identification of at-risk students and interventions to prevent them from failing, the development of more effective instructional techniques, and transforming the traditional view of the curriculum into a network of relations, using educational data collected regularly from learning management systems, social networks, learning activities, and the curriculum itself (Siemens & Long, 2011). The curriculum and its contents are part of educational data lying between the areas identified above in which big data and analytics can be used for investigation and improvement in higher education (Picciano, 2012).

### The complexity of higher medical education

The medical curriculum is inherently complex because of the multi-aspect nature of medical education (Maojo et al., 2002; Mennin, 2010). The rapidly-changing world of healthcare demands the existence of a flexible healthcare education system and consequently of a flexible medical curriculum that can be analyzed and used as a base to support and inform changes and improvements in healthcare education. Aligned with this philosophy and anticipating the need to provide a way to reduce the complexity of medical curriculum and transform it into an understandable and interoperable tool to facilitate the development and qualitative improvement of “health professions education curricula”, the Medbiquitous organization has “developed and promoted technology standards for the health professions that advance lifelong learning, continuous improvement, and better patient outcomes” (http://www.medbiq.org/about_us/mission/index.html). These technology standards use terminology in structured Extensible Markup Language (XML) format to describe the different parts of a medical curriculum.

To make this study and future studies interoperable in terms of research and benchmarking purposes, we will provide pairings of terminology used in the examined medical curriculum to Medbiquitous terminology. In this way we start from this study building a bridge between the unstructured complex medical curriculum and Medbiquitous terminology for healthcare professional’s curricula, to serve our future anticipation of standardizing the medical curriculum according to Medbiquitous standards. This will provide a transformation of medical curriculum to a structured body on which we can more easily base data-driven curriculum improvements as our effort is in this study about, and as the case is in Medbiquitous organization (http://medbiq.org/the_medbiquitous_mission).

Curriculum data used in education in the undergraduate medical program in Sweden and available to medical program and course directors, teachers, and developers (described below as stakeholders) exist in different places and in different forms and sources:

• those defined by the Swedish Higher Education Authority (higher education board) (http://english.uk-ambetet.se) and describing the intended learning outcomes (LO1–LO16 in the “Analysis and sense making of collected data” section) of the medical program at the national level;
• those on each medical program’s and course’s websites, along with the respective syllabus of the course where all learning activities, assessments, and learning outcomes are described; and
• those included in descriptions of the entire medical program at some universities in an educational database (https://internwebben.ki.se/sv/Selma).

Another source that features the same curriculum data and concerns the entire healthcare education from the highest level of the higher education board to programs and courses are those collected by medical programs prior to their external evaluations. Apart from the primary reason for external evaluation, this data was collected in an effort to transform big data from the medical curriculum into an auxiliary instrument to support education development and improvement and to create a comprehensible overview of courses and the entire medical program. Of course, the data in the form of text and numbers in various worksheets and the level of complexity are comprehensible to a certain extent to those who created that data, but they are not yet available to different stakeholders in healthcare education who only have access to curriculum data in different forms and sources as described above.

A possible use of big data in the medical education context is to:

• identify data connections and the relations between them;
• determine data’s roles in the lowest level of a course and in the overall picture of the medical program;
• perceive and analyze the curriculum in terms of identifying whether knowledge, skills, and attitude are constructed through the alignment of teaching methods and assessment towards the learning outcomes defined as constructive alignment by Biggs & Tang (2007) among different sets of data in the medical curriculum;
• perform gap analysis (Gannod, Gannod & Henderson, 2005; Ritko & Odlum, 2013) by comparing the different states in which data can be found to identify possible discrepancies and ensure the curriculum’s alignment between intended and taught curricula, not only between the different levels of the curriculum but also in the curriculum’s overall structure, towards the defined learning outcomes by Swedish Higher Education Authority.

Performing these actions on the medical curriculum is similar to performing the same actions on any complex network of information without being able to recognize the dynamics of its structure and without having adequate support for the methods and techniques applied for this purpose.

In summary, the characteristics that connect the curriculum data’s nature to big data theory within the context of undergraduate medical education and exceed the human abilities to process them easily, are:

• the complexity of both conceptual and actual structure of the curriculum;
• the large size of documents and worksheets making up the curriculum;
• the fact that curriculum is accessible from different sources and in different forms and;
• the heterogeneity of the curriculum data.

These challenges are the main factors that imply the need to find novel ways to reduce the medical curriculum’s complexity, transform it into an understandable network of information, and modify it into a flexible supportive tool in the hands of stakeholders. In this way, stakeholders could be supported in performing curriculum analysis and make decisions concerning the current and future state of medical education within a given course, easily perceiving how learning outcomes are addressed between different courses or whether there is a constructive alignment of the whole program. Additionally, stakeholders could use the benefits of a big data approach to effect changes today that would alter and improve healthcare education in the future so as to respond regularly to the changing realities of healthcare.

### Curriculum mapping

Harden (2001) defines curriculum mapping as “representing spatially the different components of the curriculum so that the whole picture and the relationships and connections between the parts of the map are easily seen”. Curriculum mapping is the appropriate way to analyze a curriculum from the viewpoint of directors, teachers, developers, and students, so that they will all be able to answer questions such as what the curriculum covers and how it is assessed, how assessment connects to teaching methods, and how students actually learn what is described in the intended learning outcomes. Substantially, this means being able to distinguish within the curriculum the different data bodies of what is taught (content of learning activities), how it is taught (teaching methods), how it is assessed (assessment), and the connections between all of them in order to achieve the intended learning outcomes. Curriculum mapping provides a strong general sense of what is important in a curriculum. Due to the idiosyncrasies of each educational system and setting and the consequent uniqueness of each educational program, curriculum mapping cannot be seen as a panacea that can simply be applied everywhere. Indeed, theory derived from curriculum mapping must be adapted to the setting of the study and the data under investigation.

### Visual analytics

**Figure 1:**
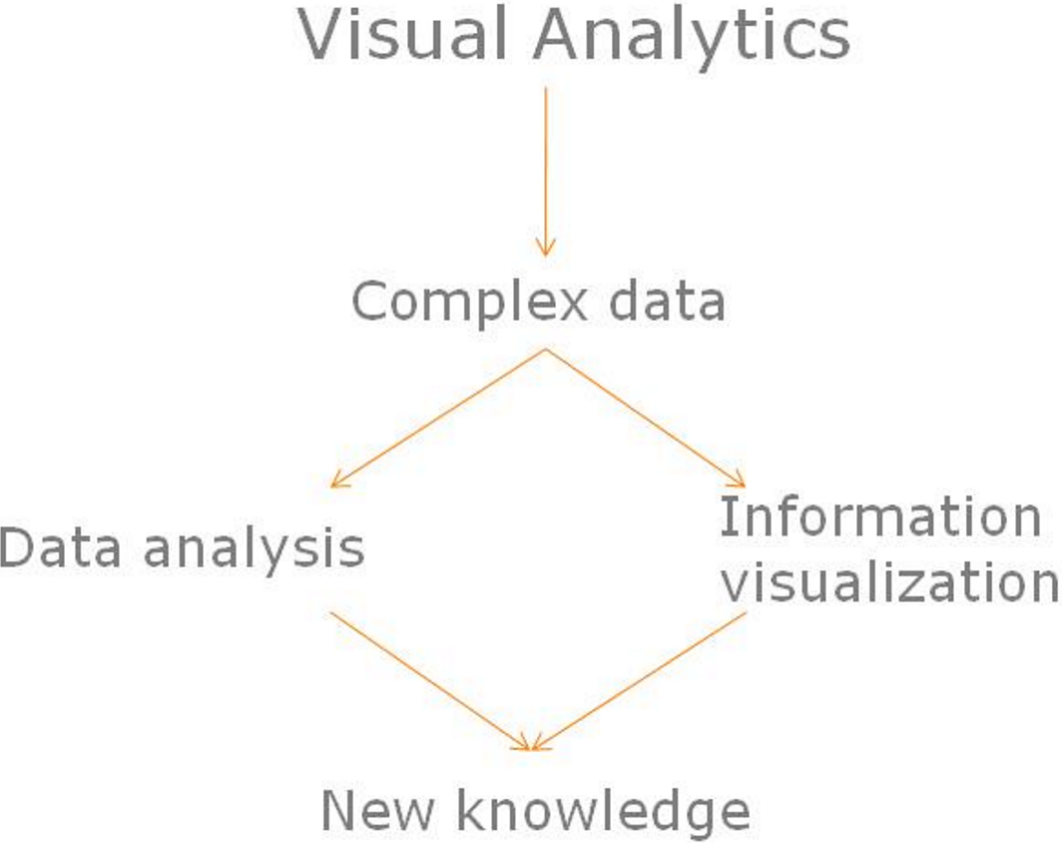
Visual analytics impact on complex data.

Methods and techniques that can manipulate data in many different disciplines have been developed (Witten & Frank, 2005; Steele & Iliinsky, 2010). Visual analytics (VA), shown in Fig. 1, is a relatively new research field that combines the two frequently used techniques of information visualization and data analysis and exploits the ability of human perception (Keim, Mansmann & Thomas, 2010). Information visualization is “the graphical presentation of abstract data” which “attempts to reduce the time and the mental effort users need to analyze large datasets” (Pantazos, 2012). The type of data analysis performed in VA is defined explicitly by the discipline being studied and the nature of the data under investigation. Data analysis, for example, can be translated into data mining techniques when analyzing and using weather data of previous years to make predictions about future weather or to adjust products in a store according to customers’ previously-recorded buying preferences (Witten & Frank, 2005). The main purpose of VA is to support the manipulation and exploitation of complicated big data, ultimately creating a holistic view of that data in order to positively impact analytical reasoning and decision-making (Keim, Mansmann & Thomas, 2010). VA permits the disclosure of previously-unknown and hidden information and patterns within the data, the use of cognitive strengths like perception and visual pattern recognition, and finally, the presentation of the processed information using visualization techniques (Keim et al., 2009; Steed et al., 2012). In the healthcare education context Olmos & Corrin (2012) have reported how analysis and simple visualization of data extracted from a healthcare education system enabled the relevant stakeholders to review instantly and preview the effects of implemented and proposed changes.

### Visual analytics in medical education

The medical curriculum is the main instrument used by different stakeholders to plan, design, and deliver healthcare education in a continuous effort to improve it. Due to its data’s complexity and the three characteristics of volume, variety, and velocity described above, it remains largely unexploited for healthcare education improvement purposes (Gathright et al., 2009). Furthermore, there is a lack of empirical data about the possible use and benefits of VA in healthcare education.

### Aim of the study

The overall purpose of this study is therefore to explore novel ways of analyzing and representing medical curriculum data using VA. The specific aims are: (i) to identify different aspects affecting how education is conducted by making sense of the collected educational data from the medical curriculum in order to determine and properly apply methods to analyze and identify the important aspects within them; (ii) to use VA to analyze further and visualize the identified aspects using a pilot course from the undergraduate medical program by assessing existing VA tools, selecting the most appropriate one and applying it; and (iii) to determine the value of applied VA methods with curriculum data.

## Materials & Methods

Exploring novel ways of analyzing and representing medical curriculum data implies the creation of an abstract model to represent the curriculum data in the initial form. Therefore this study followed the model methodology because “modeling is the purposeful abstraction of a real or a planned system with the objective of reducing it to a limited, but representative, set of components and interactions that allow the qualitative and quantitative description of its properties”. In addition, modeling methodology does not concentrate only on the model itself, but also allows the model to be used as an instrument to study the research object. It does not strictly define the modeling approach but instead is flexible, allowing the researcher to make decisions concerning the importance of various aspects of the real system that is to be modeled (Amaral et al., 2011).

### Analysis and sense making of collected data

To build a scientific basis and determine what is important to visualize within the medical curriculum, an analysis was first performed on the collected curriculum data, which is available in text format and spread across a large amount of different worksheets. It consists of different learning activities (teaching methods), assessment methods (written and other types of examinations), learning outcomes (LO1–LO16), and main outcomes (knowledge, skills, and attitude).

Below are the sixteen learning outcomes, grouped by the main outcome category to which they belong, as defined by the Swedish Higher Board of Education:

• **Knowledge and understanding**
  ○ **LO1**: Demonstrate knowledge of the disciplinary foundation of the field and insight into current research and development work as well as the links between research and proven experience and the significance of these links for professional practice;
  ○ **LO2**: Demonstrate both broad and specialized knowledge in the field of medicine and knowledge and understanding of the social circumstances that affect the health of individuals and groups, children as well as women and men;
  ○ **LO3**: Demonstrate economic and organizational knowledge of significance for the healthcare services;
  ○ **LO4**: Demonstrate knowledge of the relevant statutory provisions.
• **Competence and skills**
  ○ **LO5**: Demonstrate specialized skills in diagnosing the most frequent illnesses autonomously and in treating them in collaboration with patients;
  ○ **LO6**: Demonstrate the ability to initiate and undertake health promotion and preventive measures in healthcare services for both individuals and groups of patients;
  ○ **LO7**: Demonstrate the ability to integrate and apply knowledge critically and systematically and to analyze and assess complex phenomena, issues, and situations;
  ○ **LO8**: Demonstrate specialized skills in informing and instructing various audiences and in undertaking supervisory tasks;
  ○ **LO9**: Demonstrate the capacity for teamwork and cooperation with other professional categories in both healthcare services and health and social services;
  ○ **LO10**: Demonstrate the ability to account in speech and writing for interventions and treatment outcomes with those concerned and to document them in accordance with the relevant statutory provisions;
  ○ **LO11**: Demonstrate specialized skills in discussing new data, phenomena, and issues in the field of medicine with various audiences on a disciplinary basis and to review, assess, and use relevant information critically;
  ○ **LO12**: Demonstrate specialized skills in initiating, participating in, and undertaking improvement measures and in evaluating medical treatment.
• **Attitude (Judgment and approach)**
  ○ **LO13**: Demonstrate self-awareness and the capacity for empathy;
  ○ **LO14**: Demonstrate the ability to adopt a holistic view of patients informed by a disciplinary and humanistic approach with special consideration of human rights;
  ○ **LO15**: Demonstrate the ability to adopt an ethical and professional approach to patients and those close to them;
  ○ **LO16**: Demonstrate the ability to identify one’s personal need for further knowledge and undertake ongoing development of one’s skills.

The above data summarizes the medical curriculum from the different sources and forms that can be found and describes it separately for each course. It also describes it through an overview, moving hierarchically from higher education board to specific medical program, to courses within the program, and to different parts of a course in multipart courses. These elements of the hierarchy will be referred to below as levels in medical education and curriculum. The analysis of the collected data was also mapped to Medbiquitous standards. Therefore, references below to teaching and assessment methods will be termed events and learning outcomes and main outcomes as expectations. The simultaneous description of both events and expectations will be referred to as entities, so that each of teaching methods, assessment methods, learning outcomes, and main outcomes is an entity. Before moving forward, careful consideration was given to the recommendations of other studies outside the context of undergraduate healthcare education that use same methods in the level of the medical curriculum analysis (Corrin & Olmos, 2010; Hege et al., 2010).

After reviewing the collected data concerning the medical program as a whole, the Clinical Medicine-Reproduction and Development (CM-RD) course was selected as a pilot course for the medical program. CM-RD was chosen firstly because it is a semester-duration course resulting in 22.5 credits for students and secondly because it contains more comprehensive information than any other course within the collected curriculum data concerning all entities. It is a multipart course and includes gynecology, obstetrics, pediatrics. This study explores the course as a whole and not through its different parts; with CM-RD, the teaching methods are common, the questions on written examinations test all areas, and the learning outcomes are those defined that the students should know after finishing the CM-RD course.

From the CM-RD course one assessment method (written examination), all the teaching methods, and all the learning outcomes were selected for investigation. To analyze the above selected curriculum data and identify the important aspects that would be visualized later, the curriculum mapping framework was applied in a process described below.

### Aspects identification

Curriculum mapping (Harden, 2001) was applied to the selected curriculum data from the CM-RD course. According to that approach, when the different entities of written examination, teaching methods, learning outcomes, and main outcomes in our study and the connections between them are distinguished and highlighted, they can represent the curriculum diagrammatically, as noted above in the curriculum mapping section. The curriculum data was thus examined with an aim to distinguish these entities and the connections between them.

The information about all entities was divided into different worksheets in the curriculum data for the teaching and the assessment parts of the course. For the teaching part, all teaching methods were reviewed individually and examined for how each addresses one or more learning outcomes from the total of sixteen intended learning outcomes, thus creating a path from teaching method to learning outcome(s). For the assessment element, all written examination questions were reviewed and sorted into groups of questions that assess the same learning outcomes(s). The groups of learning outcomes were then mapped to the three main outcomes, creating another path from a written examination question and the students success rate on it to a main outcome through the learning outcome(s). Finally, all the aspects identified above were combined with an eye to the expectations to create a holistic view of the analyzed course.

Each entity’s role and how it contributes to the overall structure of the course was identified by combining the above paths from a teaching method to an assessment towards the expectations. The identification of these aspects and the appearance of clear paths between entities created a network of relations that was not visible in the primary curriculum data and allowed for determining the existence or lack thereof of the constructive alignment of the course and the performance of gap analysis. The identified aspects and the relations between them are presented in the results section.

### Assessment and selection of VA tool

A literature review was performed so as to identify appropriate tools to manipulate and visualize the identified aspects of the chosen course. However, there are no scientifically-validated VA techniques or reported appropriate tools for the analysis and visualization and representation of curriculum data. Therefore, existing tools and techniques from a plethora of open-source and proprietary software already applied for similar purposes of data visualization were investigated:

• Microsoft Excel for creating charts of different types where transfer from data to picture is supported efficiently (http://office.microsoft.com/en-001/excel-help/charts-i-how-to-create-a-chart-RZ001105505.aspx);
• Google Charts, where all popular data representation approaches from bar charts to tree-maps are available and in highly user-friendly form (https://developers.google.com/chart/);
• Gephi, which “is an interactive visualization and exploration platform for all kinds of networks and complex systems, dynamic and hierarchical graphs” (https://gephi.org/);
• BIRT, which is a web-based application that allows for the collection of data from multiple sources in order to create visualizations (http://www.eclipse.org/birt/phoenix/intro/); and
• Cytoscape, which is “a platform for visualizing complex networks and integrating these with any type of attribute data” (http://www.Cytoscape.org/).

In its early years, Cytoscape was intended to be used for biological research but it has recently been expanded to be applicable to other disciplines as well: “Although Cytoscape was originally designed for biological research, now it is a general platform for complex network analysis and visualization” (http://www.cytoscape.org/what_is_cytoscape.htm). Microsoft Excel and Google Charts were useful for this study to a certain extent, as these two tools provide a statistical character to the visualizations but they demand a unilateral investigation of the data rather than enabling both quantitative and qualitative approaches. Therefore, both were excluded from being used to visualize the curriculum data. BIRT appeared to be a promising tool since it allows the manipulation of data of significant complexity and from different sources, but BIRT requires that the data first be reformatted in order to be homogeneous and appropriate for processing by the tool. Since the intention of this study was to perform non-physical transformation on primary data from the CM-RD course, this tool was excluded from use. Gephi and Cytoscape both offered functionalities more suitable for this study, as the philosophy of these two tools is based on complex network analysis and representation.

Cytoscape was chosen over Gephi to proceed and perform the manipulation and visualization of the curriculum data for two reasons. Firstly, the programming language and environment offered in Cytoscape was already familiar to the researchers. Secondly, because the study’s goal was to transfer the underlying network, which was not obviously visible, of entities found in the primary curriculum data into a new file and build the network there for the VA tool to use without affecting the files containing the primary curriculum data. Gephi would have required intervention into the primary files, modifying them and using those altered files in the VA tool.

### Exploring the medical curriculum data with the selected VA tool

To build the network which would later be visualized, Cytoscape uses edge connections between nodes in a network. To do that simple text editors such as Notepad++ (http://notepad-plus-plus.org/) can be used to build the network row by row, which was an ideal approach for this study because it allowed for the construction of the network accordingly to the identified aspects.

**Figure 2:**
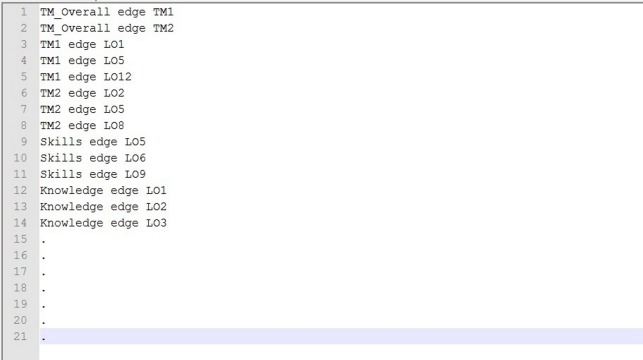
Text file containing part of the network of information before use in Cytoscape. TM, Teaching method; LO, Learning outcome.

The nodes in the data were the previously-identified entities and the edges were the connections from entity to entity. As three different networks were delimited in the “Aspects Identification” above—teaching, assessment, and their combination—three corresponding networks were built in text files for input into Cytoscape. For the teaching elements, each row of the text file was a connection from a teaching method to a learning outcome or from the total percentage of teaching methods to a single teaching method. If a teaching method was connected to more than one learning outcome, then multiple rows were used to connect this teaching method to all learning outcomes, with each learning outcome on one row. For example, one row was Teaching_Method_1—Edge—Learning_Outcome1, Teaching_Method_1—Edge—Learning_Outcome2, which corresponds to the Node-Edge-Node representation shown in Fig. 2. The same method was followed for the other two text files concerning assessment and the combination. For the assessment network, where a group of questions were used on the written examination to assess a learning outcome, a row in the input text file was a connection from this group to that particular learning outcome.

The three text files containing the networks were sequentially imported to Cytoscape, which automatically recognizes the form of the network and offers the ability to choose between different shapes and colors for nodes and edges and to move freely among and rearrange spatially nodes and edges to represent the network. Followed this method produced three different visualizations: (i) the teaching methods and learning outcomes, both taught and non-taught; (ii) the written examination questions, the learning outcomes (assessed and non-assessed), and the main outcomes, and; (iii) the constructive alignment consisting of teaching methods, groups of numbers of points on written examinations, the learning outcomes (taught and non-taught and assessed and non-assessed), the main outcomes and the results of students’ answers on written examinations in the CM-RD course.

Three different networks were created to reflect each type of available curriculum data from the CM-RD course. So, the first visualization corresponds to one file where all the teaching methods and learning outcomes of the CM-RD course are described. The second visualization corresponds to another file where the number of written examination questions, the maximum points for each question, students’ answers on the questions, and points gained and learning outcomes and main outcomes of the CM-RD course are described. The third and final visualization was created based on these two primary files, to depict how all the entities described in the files bind together in order to reveal the existence or lack thereof of the constructive alignment of the course and to perform gap analysis. This approach supported emphasizing the role of each entity and the connections between all different entities in order to allow the demonstration of both previously-perceived and non-perceived patterns and relationships within the CM-RD course curriculum data of the CM-RD course.

### Study framework

**Figure 3:**
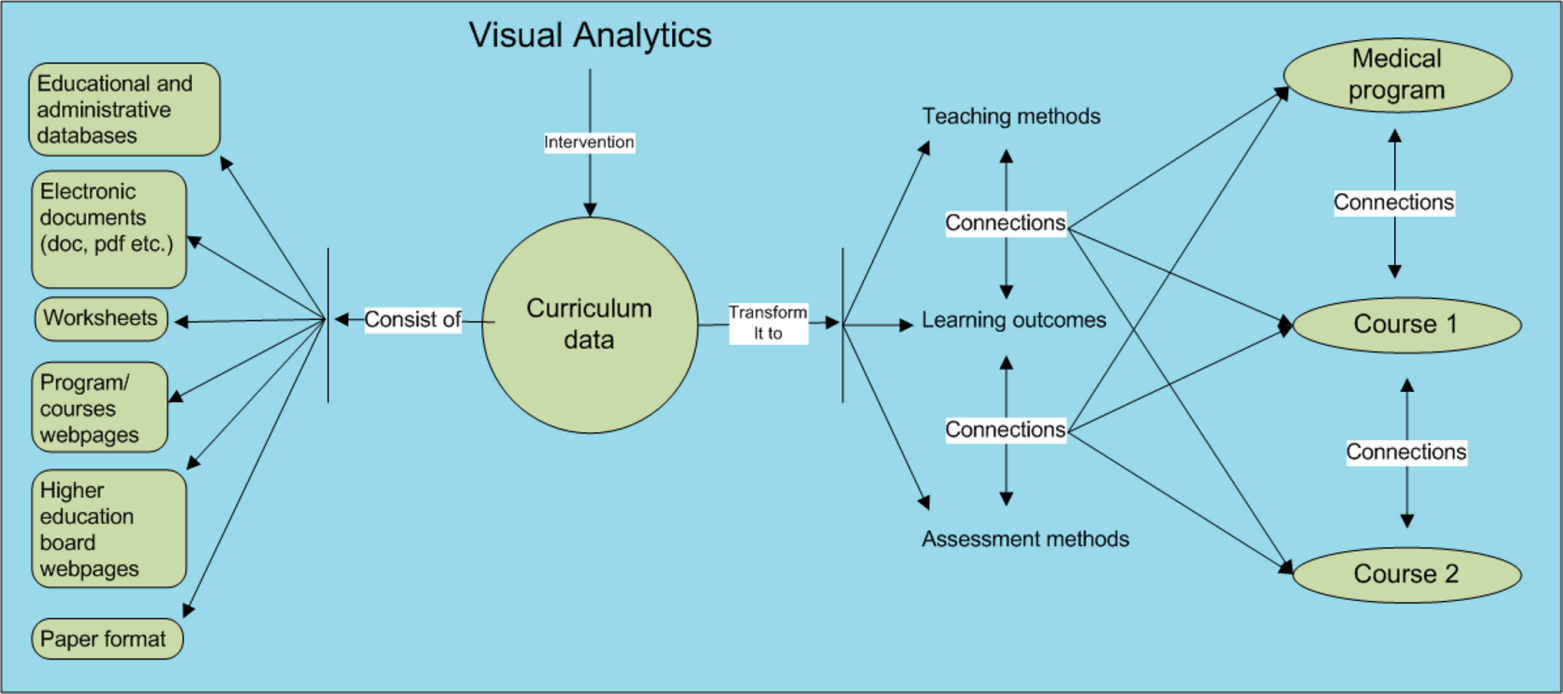
The study framework for analyzing and representing the curriculum data.

Figure 3 presents an overview of the study framework. The left side depicts how curriculum data is presently found in its different sources and forms. The large circle in the middle depicts the VA intervention into curriculum data applied in this study, while the right side shows how curriculum data is expected to appear in a structured form with connections between entities and different courses of the entire medical program made apparent after the intervention of VA.

## Results

### Identified aspects

Below are eleven aspects as they were identified in the curriculum data of the CM-RD course after they were analyzed according to the methods described above. The learning outcomes referred to in the description of some aspects of A1–11 correspond to the sixteen learning outcomes listed above. The identified aspects are:

1 The teaching methods used in the course (A1);
2 The percentages (proportions) that each teaching method is used (A2);
3 The learning outcomes taught in each teaching method (A3);
4 The percentages that each teaching method uses to address each learning outcome (A4);
5 The learning outcomes that are not addressed in any of the teaching methods (A5);
6 The proportion of questions on written examinations that assess each of the learning outcomes (A6);
7 The proportions of learning outcomes corresponding to the three main outcomes assessed on written examinations (A7);
8 The learning outcomes that are not assessed on written examinations (A8);
9 The proportions of maximum points of questions on written examinations and their relation to learning outcomes and main outcomes (A9);
10 The results of students’ answers on written examinations and their relation to learning outcomes and main outcomes (A10);
11 The connections between teaching methods, written examination, learning outcomes, and the three main outcomes (A11).

### Learning outcomes and teaching methods

**Figure 4:**
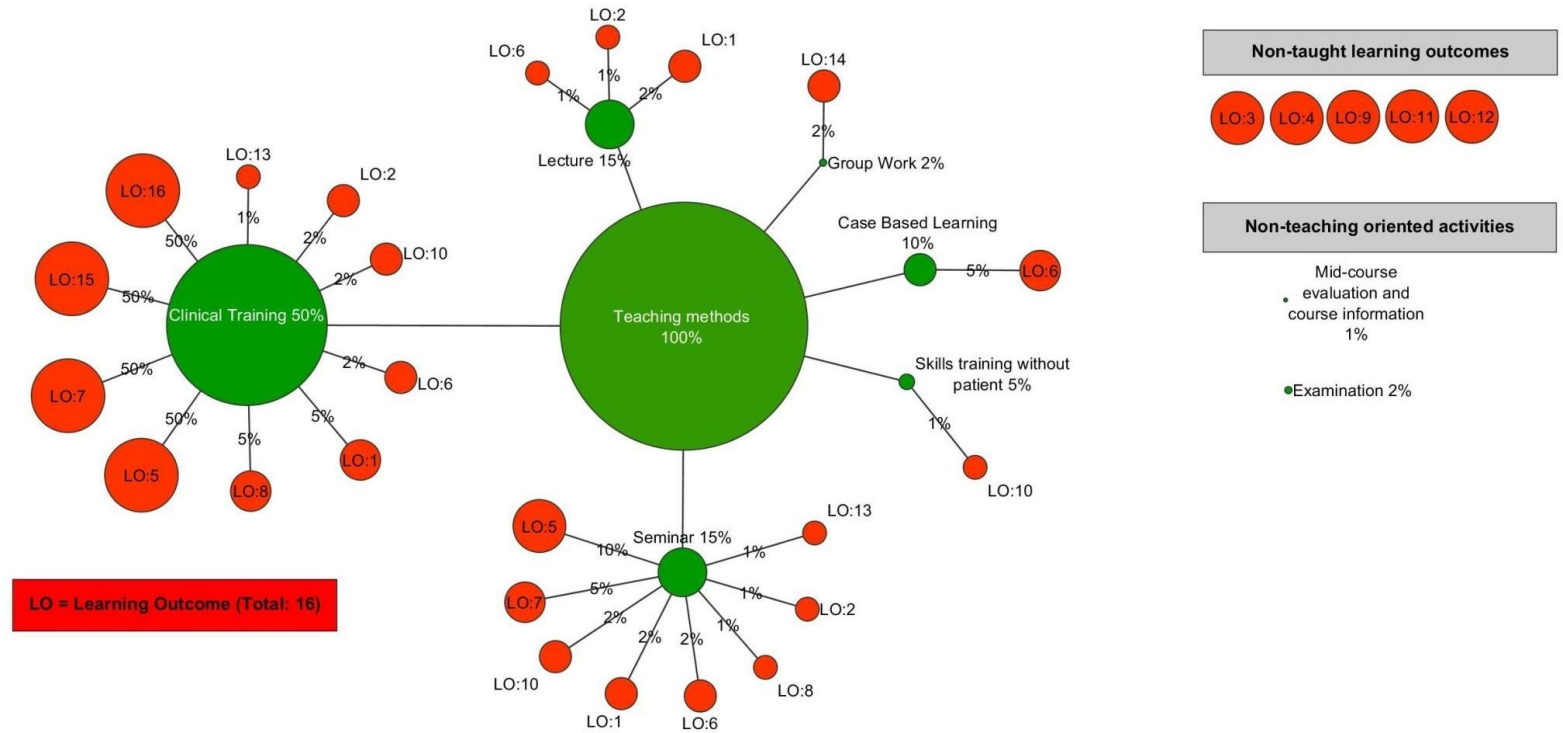
Teaching methods and learning outcomes (taught and non-taught) of the CM-RD course.

In Fig. 4, the six teaching methods of the course are depicted in green and the sixteen taught and non-taught learning outcomes are depicted in red (points A1–A5 above). The connections between the total percentage (100%) of teaching methods and the six teaching methods depict the percentages for which each teaching method is used in the course. The connections between teaching methods and learning outcomes depict the percentages of which each teaching method’s content is used to teach each learning outcome.

This visualization provides a map of learning outcomes and teaching methods in which starting from 100% of the teaching methods and going through the connections to the percentages of individual teaching methods, a hierarchy of teaching methods is created according to the relative percentages they are used. Comparing learning outcomes and teaching methods, in the event that the learning outcome’s percentage is equal to the teaching method’s percentage, this means that the teaching method uses learning activities to address this learning outcome fully. In all other cases of smaller percentages it means that fewer learning activities are used in the teaching method to address this learning outcome. For example, 2% out of 15% of lecture content is used to address LO1 (Demonstrate knowledge of the disciplinary foundation of the field and insight into current research and development work as well as the links between research and proven experience and the significance of these links for professional practice). Some learning outcomes are addressed in more than one teaching method, and in different percentages. For example, the teaching method styled “Clinical Training” uses a full 50% to address LO5 (Demonstrate specialized skills in diagnosing the most frequent illnesses autonomously and in treating them in collaboration with the patients) and teaching method “Seminar” addresses the same learning outcome using 10% out of its 15% of total learning activities. The map of learning outcomes and teaching methods is complete with the course’s non-addressed learning outcomes (top-right corner) and activities that are not teaching-oriented (middle-right).

### Examination and learning outcomes

**Figure 5:**
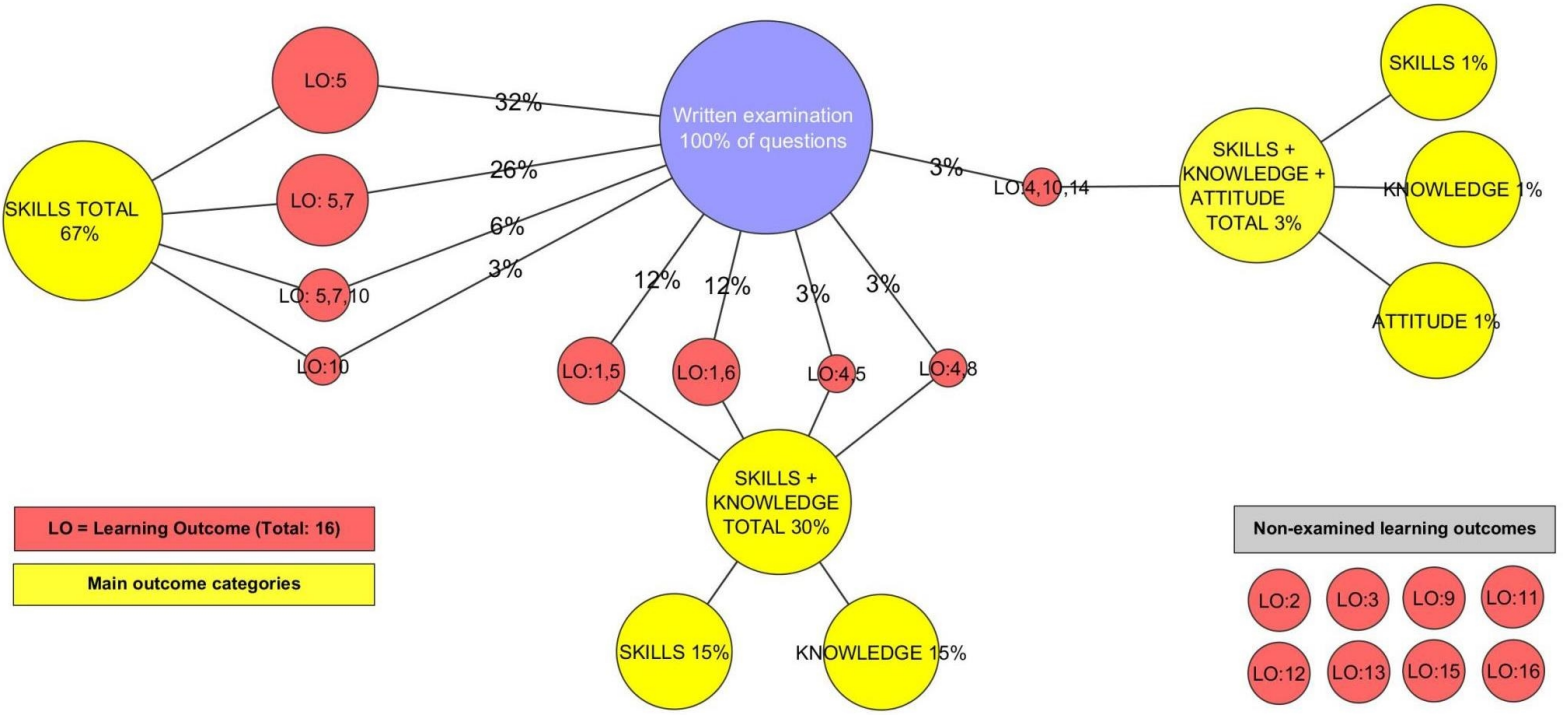
Questions in written examination, learning outcomes (assessed and non-assessed) and main outcomes of the CM-RD course.

In Fig. 5, the percentages of the total number of questions (34) used on the written examination are depicted in the connections between the blue circle (100% of the questions) and the assessed and non-assessed learning outcomes in red (points A6–A8). For example, eleven questions (32%) are used to assess LO5. The learning outcomes are connected in groups to the corresponding main outcomes, which are depicted in yellow. In cases where multiple main outcomes are assessed in groups of questions, through connections of learning outcomes the total percentage of multiple main outcomes is divided into single main outcomes. For example, 30% of the questions are used to assess skills and knowledge corresponding to 15% skills and 15% knowledge.

This visualization provides a map of how the written examination in the CM-RD course is used to assess the learning outcomes and main outcomes defined by the higher education board. In total, 83% of the questions on the written examination are used to assess skills, while 16% are used to assess knowledge and only 1% attitude. Another observation is the percentage of questions that assess each of the learning outcomes. This observation reveals how the written examination is built around the desired learning outcomes and which learning outcomes are most heavily assessed on the written examination. Some learning outcomes are assessed in more than one group of questions, like LO5 in five different cases with corresponding red circles or in combination with other learning outcomes, like LO7 in two cases.

Having available at any moment the complete non-assessed learning outcomes (bottom-right corner) and the map of the written examination concerning questions, learning outcomes, main outcomes and the connections between them, gives an easy but comprehensive overview of the written examination.

### Teaching methods, learning outcomes, examination results and gap analysis

**Figure 6:**
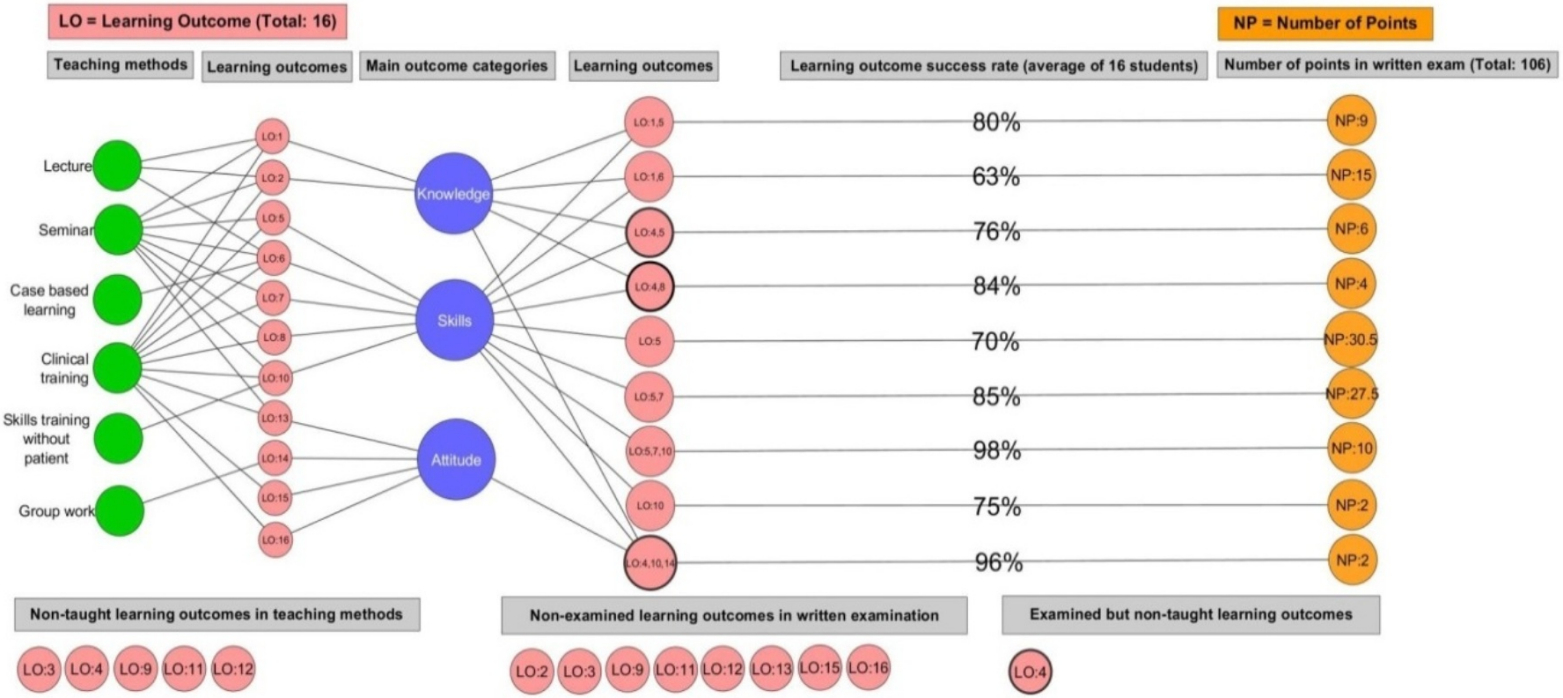
Constructive alignment and gap analysis of the CM-RD course.

In Fig. 6, the teaching methods are depicted in green, main outcomes in blue, learning outcomes—taught on the left side of the blue circles, non-taught on the bottom left side, assessed on the right side of the blue circles, non-assessed on the bottom center and assessed but non-taught on the bottom right side—in dark pink and the number of points on questions on the written examination are in orange (points A9–A11). Percentages of connections between assessed learning outcomes and the number of points depict the success rate on a learning outcome from an average of sixteen students’ answers on the written examination. The three dark pink circles surrounded with black lines on the right side of the blue circles depict the three different places that the assessed but non-taught LO4 outcome, itself on the bottom right side, can be found.

This visualization provides an overview of the whole CM-RD course with all entities from Figs. 4 and 5 and the addition of the success rate of students’ answers, all in one image. Here, the course can be observed and analyzed from different perspectives but also as an organic whole. From a teaching method to the number of points for questions on the written examination different paths exist that reveal the network of all entities. The most focuses of teaching methods and most assessed learning outcomes can be observed at the same time, revealing the trend of the course to address knowledge, skills, and attitude and to what extent in each case. The success rate of students in any of the assessed learning outcomes can be related not only to the importance of that learning outcome but also to the extent it is addressed in the teaching methods. At a glance, any gaps of taught but not assessed learning outcomes and vice versa can be identified quickly. Finally, the constructive alignment can be verified as a synthesis of possible identified gaps and the utilization of events and expectations in one place presents the course as a structured network.

## Discussion

This study strove to use VA to provide novel ways of analyzing and representing big educational data that is regularly collected for healthcare education evaluation purposes. The evaluation of different representations of one chosen course of the medical program produced with VA techniques shows that they have the potential to impact positively on perceiving entities and relations within the curriculum data. For example, gaps in learning outcomes which were not previously perceived were identified, revealing shortcomings in the constructive alignment of the examined course. Additionally, the different representations provide an overview of the course that can be used to plan and apply desired changes at present that could affect healthcare education delivery in the future.

### Analysis of pilot course

The potentials offered by VA (Keim et al., 2009; Steed et al., 2012) and presented in the results make it a promising tool to explore how the big data that is regularly collected during the evaluation of healthcare education in Sweden could contribute to the continuous improvement of healthcare education. More particularly, the enormous amounts of educational data produced in medical education in relation to teaching, learning, assessment, and outcomes and the different sources and forms these educational data can take, make it an area in which big data and visual analytics can be extremely useful to make sense of the complex information to be found in large and diverse datasets (Ellaway et al., 2014).

Since no other proposed and validated way of analyzing and representing curriculum data was found, this study cannot be related easily to other studies. Nevertheless, our findings concerning the demonstrated ability and potentials of VA and selected tools to reduce the complexity of curriculum data and make it a an understandable network of information are line with a study by Olmos & Corrin (2012) who reported how analysis and a simple visualization of data, extracted from a medical education system, allowed stakeholders to review and preview instantly the effects of implemented and proposed changes.

The rearrangement of nodes and edges representing the entities and connections was made to a modest extent intuitively and that could lead to endless efforts to represent it best, especially if an entire curriculum is to be analyzed and represented. Additionally, in the event that an entity or group of entities and connections need to be altered to apply, for example, desired changes of the curriculum to the representations, a number of static images must be created in order to be able to create a comparable before-and-after picture of the part of the curriculum that might change. Also, the interactivity allowed from produced static pictures is at low levels.

Moreover, based on the analysis of the CM-RD course, the resulting representations (Figs. 4–6) show the connections between each teaching method, questions in examination, and learning outcomes. Even if that is not the deepest level of analyzing the course, as analysis can go deeper to types of lectures, seminars, clinical training, etc., used in the teaching methods and for all parts of the course, it is complex enough to represent all these details. If all these details then need to be merged from different courses to represent the whole curriculum, the network produced, even though it will be extremely complex, will still be readily perceived.

On the other hand the rearrangement of nodes and edges resulted in different views of the same inserted network, thus giving the ability to add one very important layer to the analysis of the curriculum data. This additional layer promotes the intuitiveness of the researcher to produce different representations based on the established theory of curriculum data analysis from curriculum mapping. In this manner, the resulting representations (Figs. 4–6) indicate that this approach of analyzing and visualizing the course brings positive results and opens up a new way of looking at the CM-RD course and potentially the entire, complex medical curriculum. Even if a snapshot of the whole curriculum were used to produce the representations, they indicate additionally that they have the potential to transform the complex information in the medical curriculum into a structured and comprehensible network.

### Identified aspects of the medical curriculum

The analysis of the curriculum data of undergraduate healthcare education was based on curriculum mapping (Harden, 2001) and on the effort firstly to identify how the selected CM-RD course is structured through the connections of different entities in different levels, as there was no defined concrete series of steps that one could take that would lead to a commonly-accepted way of analyzing and representing visually a medical curriculum (Gathright et al., 2009) or even a single course in the curriculum. That resulted in identifying aspects as they are listed in the results section and building a sufficient understanding of the current structure of the course (events and expectations, http://medbiq.org/) and how all entities in it play an important role in medical education delivery and quality improvement. The VA dynamics and its possible positive impact on analyzing and representing big educational data were pilot-tested and verified on a small scale but still support conclusions for possible usage of this technique on a larger scale for more courses or the entire medical curriculum.

Additionally, the study establishes a novel way of analyzing and representing a medical curriculum which has the potential to support stakeholders to broadly analyze and make sense of it. One example is the revealed and non-previously perceived discrepancies in the delivery of the medical curriculum (Hege et al., 2010) like the learning outcome LO4 (Fig. 6) that is assessed in the written examination of the CM-RD course in three different cases but is not taught in any of the teaching methods. This approach could allow the medical education stakeholders to deliver a curriculum without gaps, thus preserving the desirable constructive alignment in the course and consequently in the curriculum.

### Representation of pilot course with VA tool

The selected tool for analyzing and representing the CM-RD course was Cytoscape. Even though Cytoscape was the most suitable, among the options explored, for the analysis and representation of curriculum data, it can produce only static images of networks, which only permits low levels of interaction with the resulting representations. It also requires substantial effort and familiarity with similar software to build the networks (Fig. 2) before they can be entered into and recognized by Cytoscape. This limitation applies even with only one course of the curriculum, as in this study.

On the other hand, a major advantage of Cytoscape is that it allows the user to create multiple representations of the same network easily and supports intuition and high levels of analysis before the choice of final representations. The potential offered by Cytoscape was considered as appropriate for this study’s purpose as it brought to light facts that were not previously perceived, like the gaps in course structure or the disproportionate way that skills, knowledge, and attitudes are assessed on the written examination. Therefore, the study establishes Cytoscape as an appropriate VA tool to represent the identified aspects of the CM-RD course in comparison to all other tested tools.

### Value of applied VA method

As noted in the introduction section, the power of VA derives from two sources. The analytics factor applied on the curriculum data through curriculum mapping aims at reducing its complexity without losing vital information and critical characteristics like the important identified aspects and relations between them; these are kept at the top level of the curriculum data map produced. The other factor is the visualization, which brought entities and relations into light by taking advantage of the human ability to process and understand visual information more easily than with raw text or in the form of a curriculum mapping report that describes the curriculum only partially.

As this study makes clear, visualization without analytics cannot stand alone and be applied to unstructured information, which makes the analytics an indispensable supporting base to a successful VA result. The analysis prior to visualization aids in structuring the inchoate curriculum data under certain norms and objectives that the visual part is then responsible for representing.

The effort required for the visual and analytics parts is not comparable and their roles are totally different. Analytics, and in our case the applied curriculum mapping method, requires substantial effort and the collaboration of different stakeholders to reshape the curriculum data from different formats and locations and bring together all the individual elements into a common format and create a network structure that represents the medical curriculum adequately. On the other hand, visualization requires less effort because the network of relations representing the curriculum data is already built. However, it requires more expertise in terms of selecting the appropriate visualization tool that has the ability to emphasize in a big picture the essential information existing in the network produced from curriculum mapping. Using the tool efficiently when transferring the network to a visualization in order to allow medical education stakeholders to perceive the network easily and perform high-level analysis cannot be accomplished without the advantage of human visual ability.

The three different representations in the results section confirm the value of VA as it was applied in this study. More specifically, Fig. 4 provides a way of analyzing the teaching part of the CM-RD course in relation to the taught learning outcomes. Using this visualization an instructor can instantly see to what extent a teaching method is used to address each of the learning outcomes in a group of learning outcomes attached to this teaching method, compare the importance of each learning outcome to the extent is addressed from the teaching method, decide if the teaching method uses the right percentages to address the learning outcomes according to previous comparisons, and support a teaching method redesign, if necessary, to be more attuned to the learning outcome’s importance. If redesigning is not necessary, then this visualization supports the instructor in evaluating and confirming instantly that the learning outcomes of a teaching method are addressed to the correct extent by this teaching method.

At any time a comparison between addressed and non-addressed learning outcomes and between used and non-used teaching methods can be performed, revealing the whole course’s map concerning teaching methods, learning outcomes, and the connections between them.

Another way an instructor can benefit from this visualization is evaluating the extent to which each teaching method is used according to the CM-RD course characteristics. As noted above, this particular course has different elements. According to the percentages each teaching method is used in total, an instructor can evaluate rapidly if the percentages of teaching methods are appropriate to address the various parts of the course and decide if an adjustment or a total redesign of teaching methods is required.

Similarly, an instructor can use Fig. 5 to evaluate how the learning outcomes of the course are assessed on the written examination. The percentages of questions can be related to the importance of the assessed learning outcome and thus suggest whether it is the correct percentage of questions, compared to the other percentages of questions used to assess other learning outcomes. The results can help the instructor decide if these percentages should be adjusted according to the importance of learning outcomes and if these learning outcomes should be assessed more fully in other types of examinations. This technique allows an instructor to discover gaps in the assessment part of the course in relation to the learning outcomes and use this visualization to modify them if necessary, creating a more learning outcome-oriented assessment.

Finally, an instructor can use Fig. 6 to evaluate the entire CM-RD course at once. Firstly, to evaluate the constructive alignment of the course, it can be used to monitor from beginning to end the path of teaching methods used to teach a learning outcome, and through the corresponding main outcome follow the path to the same learning outcome that is connected to questions on the written examination and to the points used to assess that learning outcome. Thus, an easily observed overview of how a learning outcome is taught and assessed is created and a comparison with other learning outcomes within the same visualization can take place, correlating learning outcomes’ importance and utilization in the CM-RD course so as to ensure the constructive alignment of the course.

Similarly, an instructor can evaluate, with this visualization, the entire course instantly for gaps between taught and assessed learning outcomes. For example, LO4 at the bottom-right corner in Fig. 6 exists in three different circles in the assessed learning outcomes but does not appear in the taught learning outcomes. This means that a number of written examination questions assess a learning outcome that was not actually taught in any of the teaching activities. The CM-RD course stakeholders can use this tool to analyze the course for gaps and redesign the teaching activities and examination to make them cohere. They can compare the two different course overview versions before applying these changes in reality, thus creating a more concrete and aligned course without gaps that meets the desired learning outcomes appropriately.

In sum, the difference between curriculum data analysis before VA intervention and this visualization is enormous, allowing for rapid perceiving an overview of the whole course, evaluating its constructive alignment, and performing gap analysis.

### Strengths and limitations

The main strength of the study was the access to genuine educational data currently used in medical education. The data were prepared for review from the Swedish Higher Education Authority, summarized the medical curriculum and after being reviewed, were considered appropriate to conduct this study. The model methodology followed in this study also added flexibility in terms of deciding which aspects of the medical curriculum should be modeled and visualized to create the final model.

On the other hand, this study was limited to analyzing just one course’s structure and particularly the teaching methods, the written examination, and the relation between them and to the learning and main outcomes.

### Implications for healthcare education

The findings of this study contribute to the medical education field with new knowledge using VA. They also open a new area for investigation in the medical education informatics field. The VA approach used in this study to analyze and represent the CM-RD course through the different representations appears to have good potential to:

• reveal the hidden structure of curriculum data examined between different entities;
• identify gaps and roles of minor–major entities in the structure of the data;
• possibly be used as an instrument for planning and applying future changes to the curriculum in the present, in an effort to be able to align medical education constantly with the changeable demands of the healthcare setting.

Generally, the contribution of this study resides in the novel ways that it provides for verifying ongoing medical education structure and analyzing and deciding about design and planning of activities in the hands of teachers and directors to support medical education quality improvement.

Since the Swedish Higher Education Authority defines the learning outcomes for undergraduate medical education at the national level, it would be promising to apply the same VA techniques presented in this study to medical education in other locations in the country and investigate possible similarities and differences among them in terms of the study objectives. However, to reach the point that medical curricula from different regional universities can be compared even at the level of a pilot course, significant preparatory work must be undertaken. As noted in the introduction, the data used in this study summarizes the material from the medical curriculum as it exists in different places and forms. This means that the preparatory work from unstructured to structured—but still complex and unexploited for medical education improvement purposes—curriculum data, had already been done. This preparation requires devoting time, resources, and effort and applying expertise to produce data that can be further analyzed and presented with VA. This necessity adds another “thick” layer of processing raw big educational data but as this study demonstrates, the added value of using VA likely constitutes more than enough justification to undertake such initiatives.

The fact that VA techniques must be applied in the data used in this study implies that, even if it is not in the unstructured form as primary curriculum data, it remains big educational data, even if it would not be in another category. As analyzed above in the general discussion about big data, the three characteristics that coexist in data within a system or domain are enough to challenge constraints to manipulate and analyze it so that it can be used to good effect. Within this system or domain the data is considered big data irrespective of whether it might be considered small when compared to another domain. Depending on the domain the volume of data can vary from megabytes to petabytes. For example, a 40 MB presentation is considered big in comparison to the typical size of a PowerPoint presentation. Thus, big data may refer to different sizes and types from domain to domain but all these domains share a common challenge that must be addressed, which is being able to search, analyze, and make sense of the data (Zaslavsky, Perera & Georgakopoulos, 2013).

The separation in different categories of big educational data is derived from the fact that techniques that applied to the data of this study cannot be applied to the primary data and vice versa. Without the use of special techniques to analyze and represent the summarized data, it would remain unexploited as it has been until today for medical education improvement purposes, because of its complexity. Different techniques are applied for different purposes and primary data must be formatted to be processed further with VA, using different techniques than the one demonstrated in this study. Additionally, this study has the potential to be generalized outside Swedish boundaries. The current data was analyzed partially based on existing curriculum mapping framework that has been used widely in higher education in general and more extensively in medical education.

### Future research

The findings of this study suggest that further investigation is required to both reduce the complexity of the whole medical curriculum and to delve more deeply into the different parts of multipart courses to create a holistic view of medical education and be able to draw conclusions that can improve it from a more general viewpoint. To achieve this goal, novel and more interactive ways of representing the curriculum with more details while still reducing its complexity must be investigated. This involves investigating new tools that are able to perform such actions or even the creation of customized tools for these purposes. Additionally, the current approach must be adjusted to analyze and represent multipart courses in healthcare education with more details, again without increasing the complexity of representations to unacceptable levels.

## Conclusions

In this study we used curriculum mapping to make sense of collected curriculum data from an undergraduate medical program. This made it possible to analyze the data and identify important aspects that affect how medical education is conducted. Through the assessment of existing VA tools, no validated VA tool for analysis and visualization of curriculum data was found and therefore the most appropriate one was selected to build an abstract model of the examined data in three different approaches: (i) learning outcomes and teaching methods, (ii) examination and learning outcomes, and (iii) teaching methods, learning outcomes, examination results, and gap analysis. The level of analysis offered by these three approaches allow the user to create novel, penetrating insights and an understanding of the underlying information in the curriculum data that was not possible before the use of VA. More generally, the value of the VA method applied in this study resides in the promotion of analytical reasoning in order to create both a detailed understanding and a high-level overview of the examined data, to master past and present situations and support decision-making for future situations.

In summary, the findings of this study provide a novel way of evaluating and verifying how ongoing medical education is structured by perceiving the role of learning and assessment activities towards the desired learning outcomes in the big picture of the curriculum, and foster planning and designing with a solid understanding of the core information in the curriculum with VA as a tool with possible positive implications on medical education informatics research and on how quality improvement of medical education is designed.

## Supplemental Information

10.7717/peerj.683/supp-1 Figure S1 Teaching methods Teaching methods and learning outcomes (taught and non-taught) of the CM-RD course.

10.7717/peerj.683/supp-2 Fgiure S2 Assessment Questions in written examination, learning outcomes (assessed and non-assessed) and main outcomes of the CM-RD course.

10.7717/peerj.683/supp-3 Figure S3 Constructive alignment Constructive alignment and gap analysis of the CM-RD course.

10.7717/peerj.683/supp-4 Appendix S1 Appendix S1 Description of the sixteen learning outcomes and main outcome categories they belong.
