# Supplementary figures and images for: Visual analytics in healthcare education: exploring novel ways to analyze and represent big data in undergraduate medical education
Source: PeerJ. 2014 Nov 25;2:e683. doi: 10.7717/peerj.683 (PMC4250066; doi:10.7717/peerj.683)

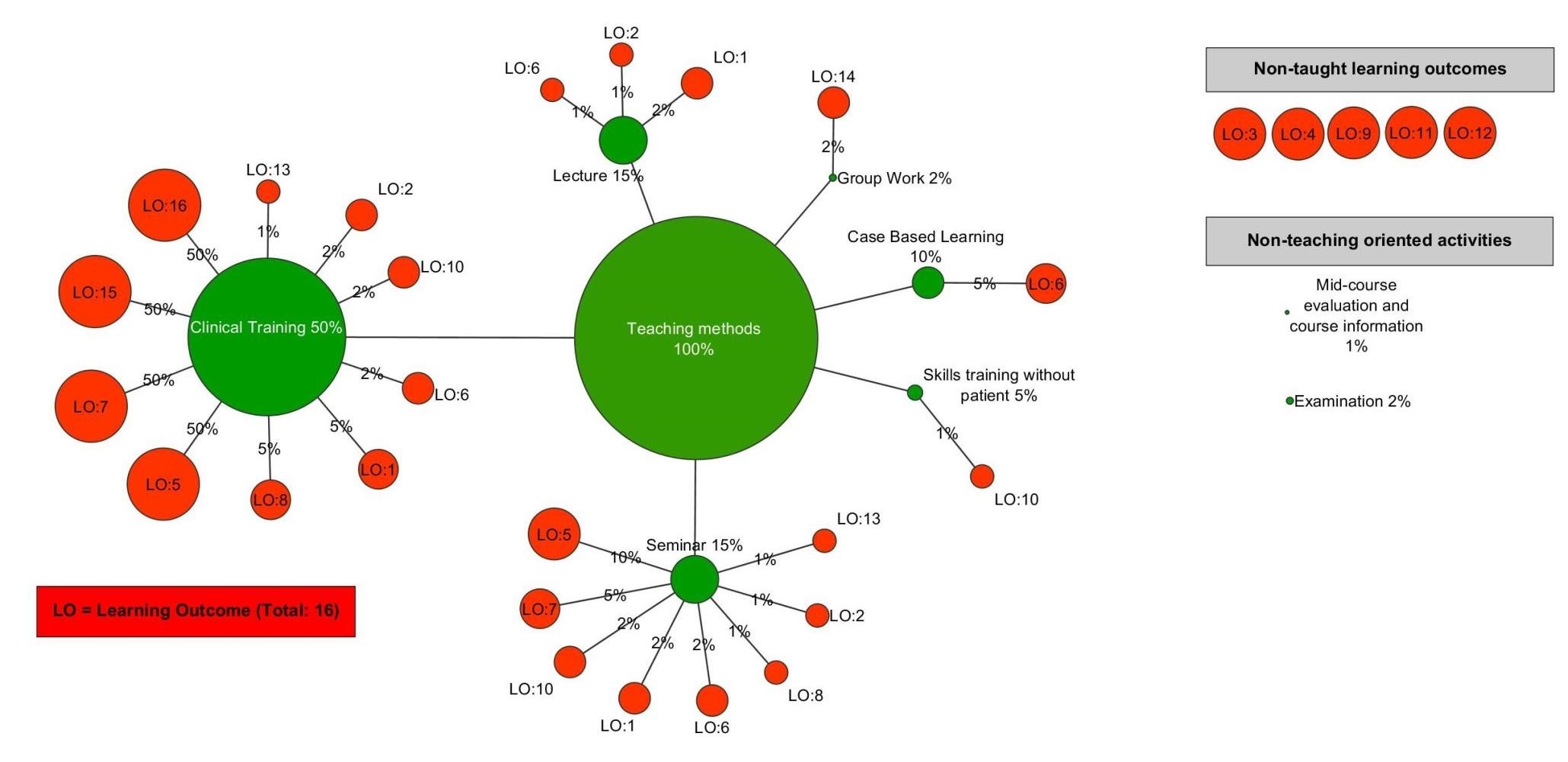

Supplement: Figure S1 — Teaching methods and learning outcomes (taught and non-taught) of the CM-RD course. [file peerj-02-683-s001.jpg]

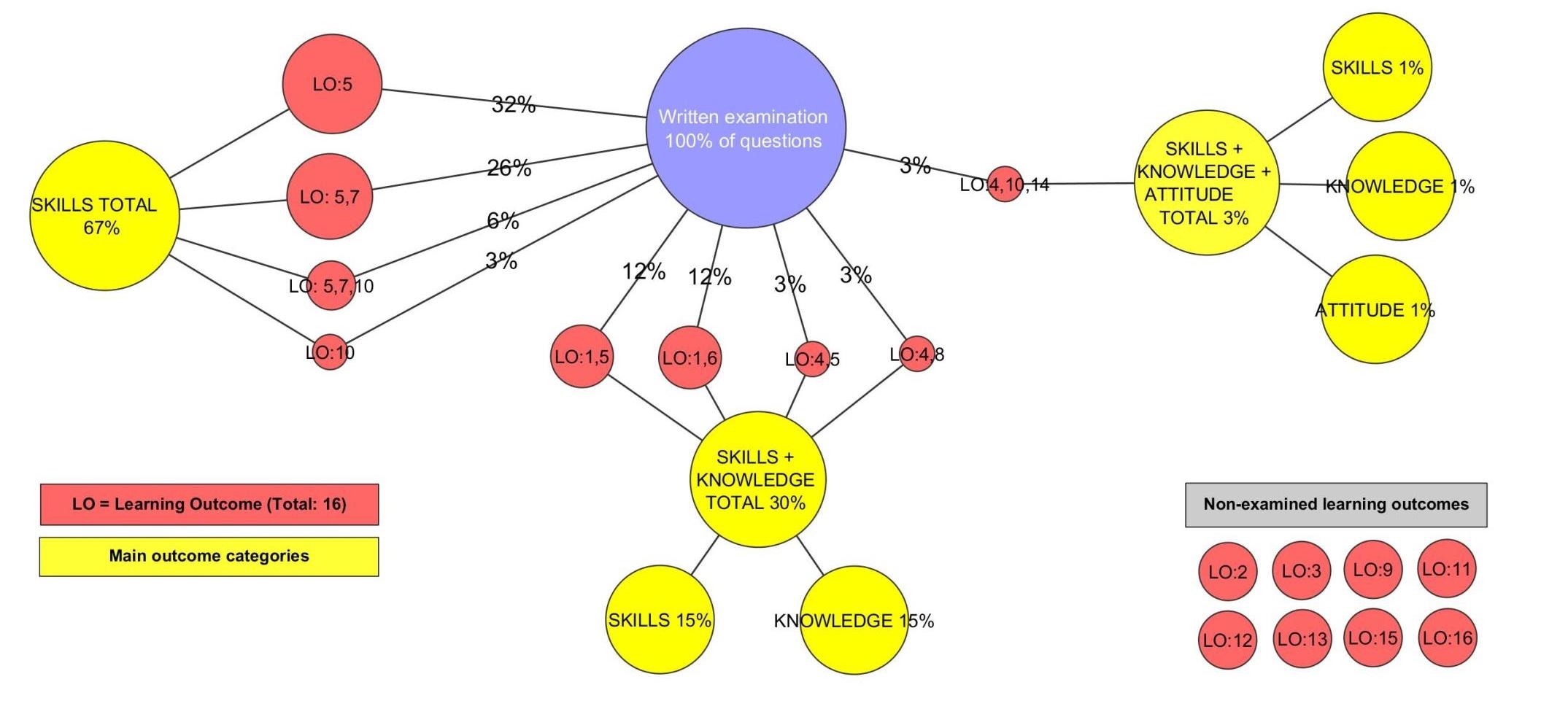

Supplement: Fgiure S2 — Questions in written examination, learning outcomes (assessed and non-assessed) and main outcomes of the CM-RD course. [file peerj-02-683-s002.jpg]

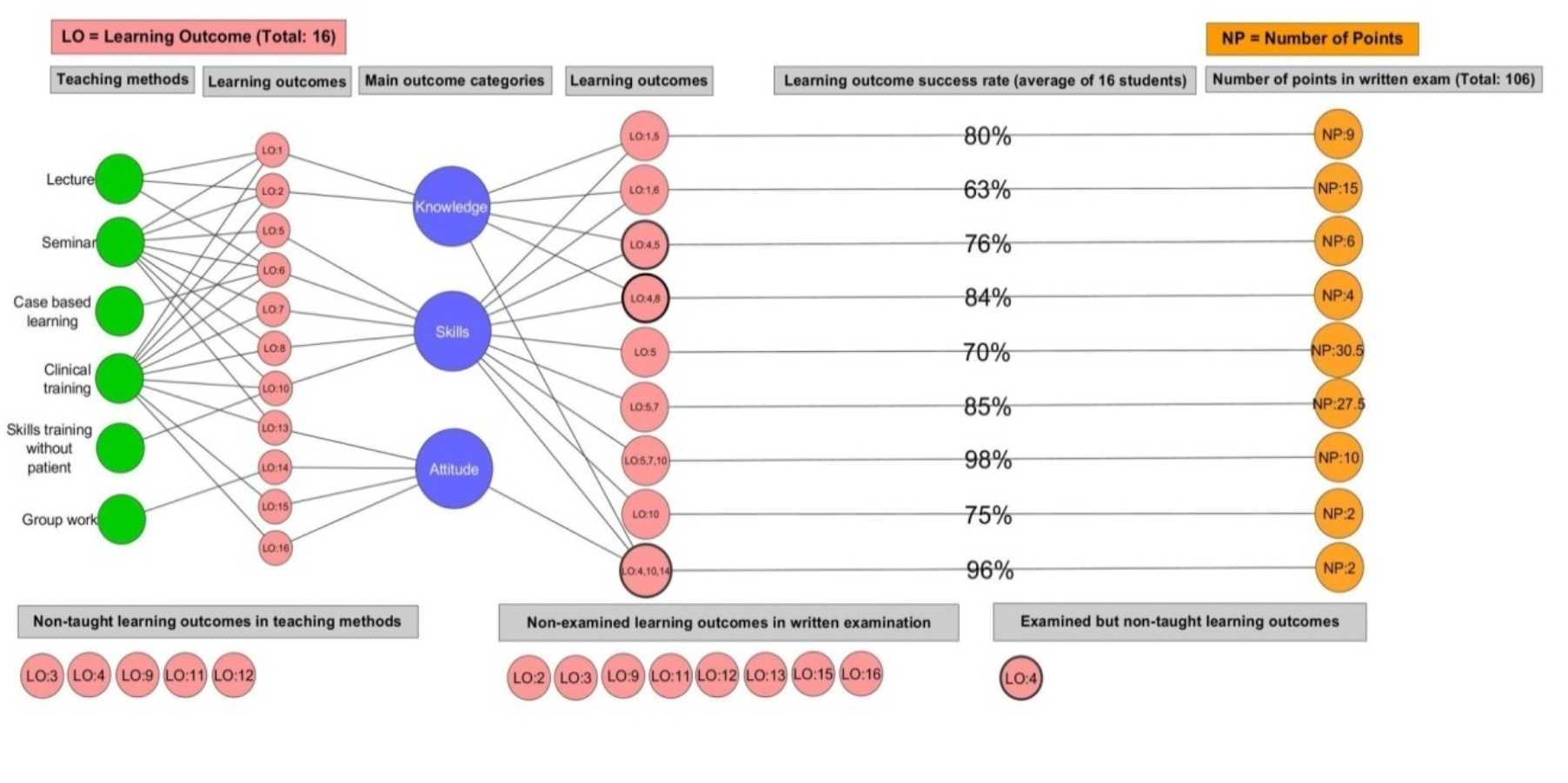

Supplement: Figure S3 — Constructive alignment and gap analysis of the CM-RD course. [file peerj-02-683-s003.png]
