# Supplementary material for: Visual analytics in healthcare education: exploring novel ways to analyze and represent big data in undergraduate medical education
Source: PeerJ. 2014 Nov 25;2:e683. doi: 10.7717/peerj.683 (PMC4250066; doi:10.7717/peerj.683)
Supplement: Appendix S1 — Description of the sixteen learning outcomes and main outcome categories they belong. [file peerj-02-683-s004.docx]

Appendix A. Description of the sixteen learning outcomes and main outcome categories they belong

|  | **Short description** | **Main outcomes** |
| --- | --- | --- |
| LO1 | Demonstrate knowledge of the disciplinary foundation of the field and insight into current research and development work as well as the links between research and proven experience and the significance of these links for professional practice |  |
| LO 2 | Demonstrate both broad and specialized knowledge in the field of medicine and knowledge and understanding of the social circumstances that affect the health of individuals and groups, children as well as women and men | Knowledge  and  Understanding |
| LO 3 | Demonstrate economic and organizational knowledge of significance for the health care services |  |
| LO 4 | Demonstrate knowledge of the relevant statutory provisions |  |
| LO 5 | Demonstrate specialized skills in diagnosing the most frequent illnesses autonomously and in treating them in collaboration with the patients |  |
| LO 6 | Demonstrate the ability to initiate and undertake health promotion and preventive measures in the health care services for both individuals and groups of patients |  |
| LO 7 | Demonstrate the ability to integrate and apply knowledge critically and systematically and also to analyze and assess complex phenomena, issues and situations |  |
| LO 8 | Demonstrate specialised skills in informing and instructing various audiences and also in undertaking supervisory tasks | Competence  and |
| LO 9 | Demonstrate the capacity for teamwork and cooperation with other professional categories in both the health care services and the health and social services | Skills |
| LO 10 | Demonstrate the ability to account in speech and writing for interventions and treatment outcomes with those concerned and to document them in accordance with the relevant statutory provisions |  |
| LO 11 | Demonstrate specialized skills in discussing new data, phenomena and issues in the field of medicine with various audiences on a disciplinary basis and also to review, assess and use relevant information critically, and |  |
| LO 12 | Demonstrate specialized skills in initiating, participating in and undertaking improvement measures and also in evaluating medical treatment. |  |
| LO 13 | Demonstrate self-awareness and the capacity for empathy |  |
| LO 14 | Demonstrate the ability to adopt a holistic view of patients informed by a disciplinary and humanistic approach with special consideration of human rights | Judgement  and |
| LO 15 | Demonstrate the ability to adopt an ethical and professional approach to patients and those close to them, and | Approach |
| LO 16 | Demonstrate the ability to identify the personal need for further knowledge and undertake ongoing development of his or her skills. |  |
